# Interview-based sighting history to investigate the historical range and dynamics of dugongs in China

**DOI:** 10.1098/rsos.250486

**Published:** 2025-10-22

**Authors:** Mingli Lin, Yuanyuan Li, Yifei Cai, Haozhong Chen, Zirui You, Ouhoud Soufiane, Songhai Li

**Affiliations:** ^1^ Marine Mammal and Marine Bioacoustics Laboratory, Institute of Deep-sea Science and Engineering, Chinese Academy of Sciences, Sanya 572000, People’s Republic of China; ^2^ University of Chinese Academy of Sciences, Beijing 100049, People’s Republic of China; ^3^ The Innovation Research Center for Aquatic Mammals, and Key Laboratory of Aquatic Biodiversity and Conservation of the Chinese Academy of Sciences, Institute of Hydrobiology, Chinese Academy of Sciences, Wuhan 430072, People’s Republic of China

**Keywords:** dugong, marine mammals, extinct, ecological knowledge, citizen science

## Abstract

The dugong (*Dugong dugon*) is the first marine megafauna to be declared functionally extinct in China, yet its historical range and extinction dynamics remain poorly understood. Sighting histories were thus collected from 841 fishers to investigate this information based on a large-scale interview survey across the entire dugong historical range in 2024. Apart from a single incidental capture reported in 2021, there have been no other records reported by informers since the last stranding in 2008, with the average date of the last sighting being in 1983 ± 14. A notable discovery was that dugongs were once sighted in Shantou, extending the known historical range northward by 500 km in mainland China. We also document extensive past dugong sightings and seagrass meadows in the South China Sea, beyond the previously known range. The spatio-temporal analysis indicates that dugongs disappeared almost simultaneously across their entire historical range, without undergoing significant range contraction. These findings confirm previous conclusions that dugongs are now functionally extinct in China. Our study reveals how a once widely distributed marine mammal experienced a population crash within just 20–30 years, serving as a serious warning for dugong conservation worldwide and highlighting the urgent need to protect other marine megafauna in the South China Sea.

## 1. Introduction

Effective conservation of endangered species relies on a thorough understanding of geographical range and spatio-temporal dynamics. Geographical range contraction is generally considered as a critical indicator of population decline, often serving as an early warning of progression towards extinction [1,2]. Investigating biogeographic patterns, temporal rates and ecological drivers of range collapse across various species groups and regions is thus essential for developing effective conservation strategies. These studies can provide invaluable insights into the environmental pressures affecting species and inform targeted mitigation efforts, particularly in addressing habitat loss and fragmentation. However, the dynamic biogeography of range collapse remains largely underexplored [3]. Most research has concentrated on documenting or estimating historical range contractions for specific species, rather than examining ongoing processes [4]. Bridging this gap could lead to significant advancements in conservation approaches, enhancing efforts to protect and recover threatened species.

Research on endangered species’ geographical distribution and range collapse often presents significant challenges. These species typically have low population densities, which greatly limits the applicability of standard visual-based field survey [5,6]. This issue is pronounced for endangered mammals with typically broad distributions, making it a big challenge to understand their geographical and spatio-temporal patterns [7]. For many developing countries, the challenges are compounded by a lack of funding and trained researchers [8], which often results in the absence of systematic population ecology studies, even after a species has severely declined or gone extinct. In such cases, the studying of historical range and collapse processes requires approaches that extend beyond traditional field-based surveys. With the rise of citizen science, feasible methods for large-scale monitoring of the spatial and temporal distribution dynamics of endangered and rare species have emerged [9,10]. The acquisition and use of local ecological knowledge (LEK), which represents experiential knowledge developed by resource users through interactions with local environments, is a commonly employed approach in citizen science to assess population status [11–14]. Although LEK is often criticized for its limitations and potential biases, it provides valuable insights and complements traditional methodologies, particularly for rare or cryptic species which are otherwise challenging to study using standard field survey methods [6,7,15]. However, its application to studying the historical distribution and extinction dynamics of marine megafauna remains limited.

**Figure 1.**
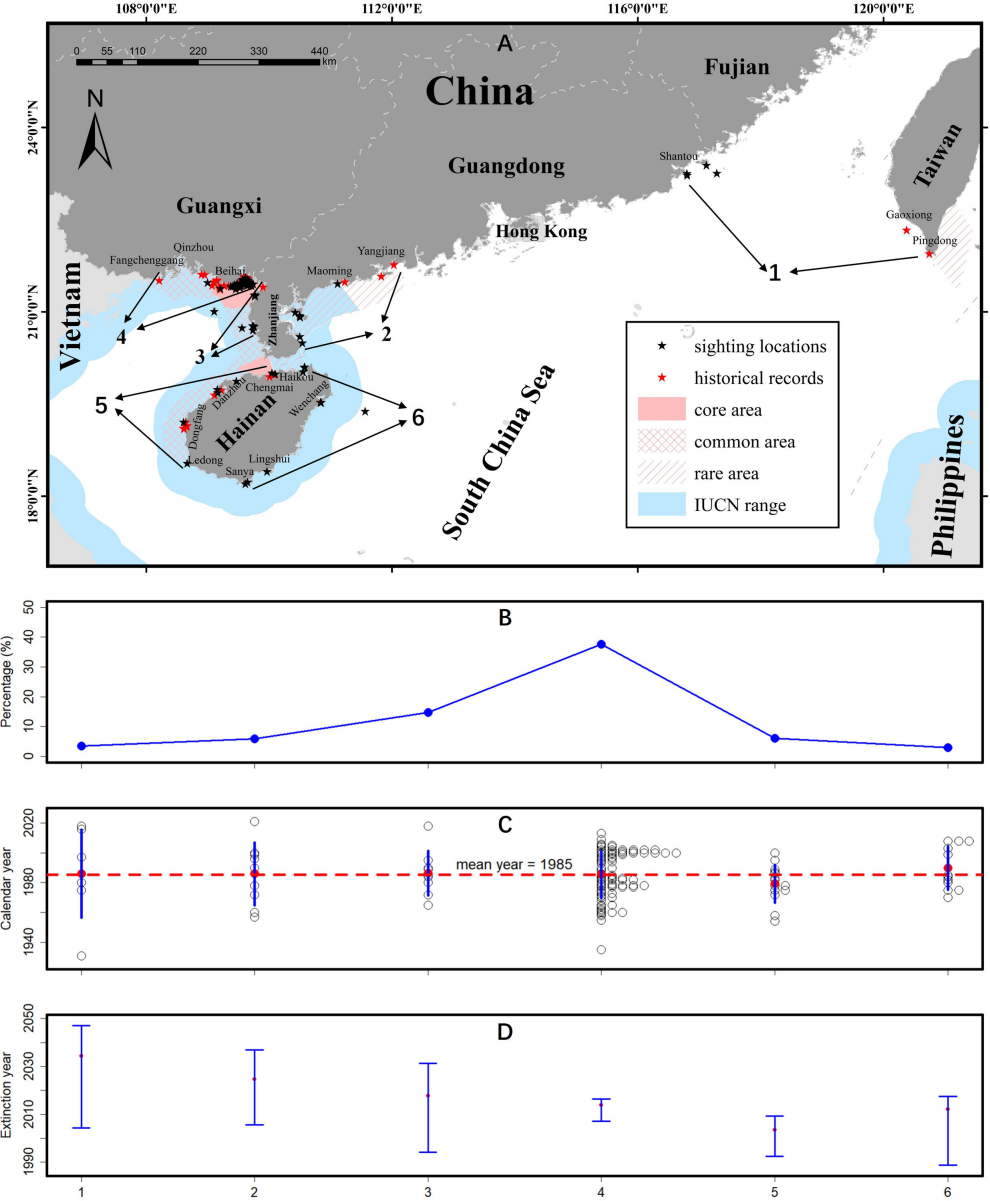
(A) Distribution of dugong in Chinese waters. (B) Percentage of informants fishing in six different areas who have reported dugong sightings. (C) Temporal distribution of dugong last sighting dates in different areas. The red dashed line indicates the average last sighting date. (D) Mean and 95% confidence intervals for optimal linear estimation of regional dugong extinction date in different areas.

A recent study indicates that dugongs (*Dugong dugon*) are now functionally extinct in Chinese waters, suggesting that the population has experienced a permanent failure of reproduction or recruitment [16,17]. This was the first documented case of a large vertebrate functional extinction in Chinese marine waters. While dugongs have been recorded in China for several centuries [18], scientific study only began in the mid-twentieth century on morphology [19,20] with historical range remaining largely unknown due to limited field surveys. Wang & Sun conducted the first investigation of dugong distribution in China [21]. Based on several field studies conducted primarily in the 1980s, combined with museum specimen analyses and internal data reviews, they identified the Guangxi coast in the Beibu Gulf, the western Leizhou Peninsula in Guangdong and the northwestern waters of Hainan Island as the range of dugong in China, while also noting southern Taiwan as the northern limit of distribution (figure 1A). Following this, only two scientific surveys specifically focused on dugongs in Chinese waters. Zhou *et al.* conducted 36 boat surveys along the Beibu Gulf coast to assess dugong status, with a group of five individuals near Gangmen Village in Dongfang recorded on 8 September 2000 [22]. However, this record was later challenged by another survey in the same area. Wang *et al.* conducted interviews in the Beibu Gulf and carried out a year-long visual survey in 2002 in western Hainan coastal waters [23]. Their findings suggested dugongs still existed near Hepu, Guangxi, but none were found in coastal waters of western Hainan. They concluded that the likelihood of observing a group of five dugongs in Dongfang waters was very low due to seagrass bed destruction. No surveys on Chinese dugongs were conducted afterwards until a questionnaire-based survey in 2019 covering historical range confirmed functional extinction [16]. The lack of research on the historical range and geographical distribution dynamics has severely limited understanding of the extinction process. Insights into this process could not only inform conservation efforts for other dugong populations worldwide but also provide valuable experience for protecting marine megafauna in China.

To address this knowledge gap, we conducted an interview survey in 2024 to investigate the historical distribution of dugongs in China. Additionally, we use Chinese dugongs as a case study to investigate how a widely distributed marine megafauna can rapidly go functionally extinct, aiming to enhance our understanding of the spatio-temporal range dynamics during extinction processes.

## 2. Method and material

### 2.1. Interview survey

We conducted fieldwork to investigate the current and past status of dugongs and regional fisheries between 10 July and 13 August 2024, during the mandatory fishing ban. This timing was advantageous, as many fishers were at home, facilitating the collection of a sufficient sample size. We employed standard LEK interview techniques, which have been previously applied in marine megafauna research in the South China Sea [24,25]. Interviews were carried out in fishing communities across all 11 coastal provinces of mainland China. However, given that historical records of dugongs are limited to provinces south of Taiwan, we focused our survey on Fujian, Guangdong, Guangxi and Hainan. These four provinces encompass the entire historical range of dugongs in the Chinese mainland [16]. In total, 841 responses were collected from 54 villages across 27 cities in these four provinces. The sampling design was informed by data on the number and locations of fishing ports provided by the China Fishery Statistical Yearbook [26]. According to the yearbook, the provinces of Fujian, Guangdong, Guangxi and Hainan have 15 464, 32 758, 6779, 22 349 registered fishing boats, respectively. Based on a 1% proportional sampling strategy, we conducted 194 (1.25%), 329 (1.00%), 80 (1.18%) and 238 (1.06%) interviews in these four provinces.

Given the extreme rarity of dugongs in Chinese waters, we carefully selected informants to maximize the likelihood of reliable observations [14]. Only professional fishers were interviewed, while informants from coastal communities, such as coast guard personnel, cargo ship crew or ferry drivers, whose livelihoods may even have a close connection with the coastal ecosystem, were excluded. This is based on the consideration that fishers typically have the most direct and long-term interaction with the marine environment; thus, they are the most likely group to have observed rare or elusive species such as dugongs and to possess detailed knowledge about historical presence, habitat use and population trends. Fishers eligible for the survey had to be at least 18 years old and have lived in the survey area for a minimum of 5 years. We did not impose selection criteria based on adult age or years of fishing experience. While older fishers offer valuable historical insights from earlier decades, younger fishers are more likely to report recent sightings, which are essential for assessing the current population status and the potential presence of remnant individuals. Respondents were chosen through random encounters within fishing communities, with no selection criteria based on sex, ethnicity, income, education or other demographic characteristics. The interviews were conducted by a team of five researchers with expertise in marine mammals, supported by 17 trained volunteers recruited from universities, most of whom were studying biology or marine ecology. Volunteers underwent 2 days of training focused on dugong identification and interview techniques before participating in the fieldwork. The first completed questionnaire for each volunteer was carefully reviewed by an experienced researcher, who provided feedback on any issues and excluded it from the dataset. During the survey process, any inadequately completed questionnaires were also discarded and not included in the data analysis. All interviews were conducted only after obtaining verbal consent from participants, who were assured that responding information would remain anonymous and be used exclusively for scientific research.

As part of a broader series of interview questions, informants were asked about age, the number of years and days per year they typically spent fishing, and whether they had seen a dugong. They were also requested to provide the date, exact location and behaviour of the most recent dugong sighting, as well as any other related knowledge. Additional questions included whether seagrass was present in the areas where dugongs were sighted, the distribution of seagrass, when seagrass coverage began to decline, and the reasons for this decline. Informants were also asked if they had ever hunted dugongs, including the year of hunting and the purpose. All informants were required to identify, without prompting, photographic cue cards depicting both live and dead dugongs to test accurate identification of the species and validate responses. When informants reported a dugong sighting, we asked them to provide the local name and describe distinguishing features compared with other large marine vertebrates to verify correct species identification.

### 2.2. Data analysis

Given the difficulty of obtaining sighting data for endangered marine mammals, we referred to previous methods used for collecting baiji (*Lipotes vexillifer*) sighting data and obtained reliable last sighting records through multiple sources [3]. In addition to 52 records with last sighting years collected from the 2024 questionnaire data, we included sighting records (*n* = 37) with specific last sighting years (*n* = 23) obtained from a previous fisher questionnaire survey in 2019 to comprehensively reflect the distribution of dugong in China. The methods used to collect these additional data in 2019 are detailed in our previously published work [16]. Furthermore, 59 historical records that included sighting years were also used to analyse the temporal range of dugong last sighting dates and to perform optimal linear estimation (OLE) for assessing regional extinction dates in different areas. Since the three data sources are not entirely consistent, they were used only to analyse the historical distribution of dugongs and the temporal trends in sighting records.

Although most reported dugong sighting dates (40%) were given as specific calendar years, many informants provided last sighting dates in alternative formats. These included paired consecutive years (e.g. ‘1965/1966’), decadal or other ranges (e.g. ‘1970s’, ‘late 1970s’, ‘1980−1985’), approximate time periods relative to 2024 (e.g. ‘30 years ago’) or approximate values (e.g. about ‘1965’) and other formats (e.g. when I was 10 years old). Given that most sightings occurred a long time ago, relying solely on reports with exact calendar years would result in significant information loss. To incorporate this additional data into further analyses, alternative formats were converted into direct calendar years based on the method provided by Turvey *et al* [3]. For paired consecutive years, sighting dates were randomly assigned with equal probability to either year. Decadal ranges or specific year ranges were assigned with equal probability to any year within the range. Dates rounded to a certain number of decades or half-decades before 2024 were assigned an equal probability across a ±5 year range around the specified value (e.g. ‘30 years ago’ represented a potential date range from 1989 to 1999). Approximate values were also assigned an equal probability for any year across a ±5 range. Other formats will be converted directly to direct calendar years. Only data with completely indeterminate dates, such as ‘decades ago’ or ‘long time ago’, were excluded.

To analyse the spatio-temporal dynamics of dugong sighting history in different areas, we divided the study region into six zones (figure 1A). This division was primarily based on differences in coastal environments and geographic locations. The Beibu Gulf features slow depth variation and extensive shallow-water areas suitable for seagrass growth. This region was further subdivided by province into three zones: zone 3, located off the western coast of Guangdong’s Leizhou Peninsula; zone 4, along the Guangxi coast; and zone 5, off the western coast of Hainan Island. By contrast, zones 1, 2 and 6 are in the open waters facing the South China Sea, where depth changes occur more rapidly and seagrass is restricted to nearshore intertidal zones and shallow waters [27–29]. Based on historical dugong sighting records, zone 1 encompasses eastern Guangdong and southern Taiwan, zone 2 covers the western coast of Guangdong’s Leizhou Peninsula and zone 6 includes the eastern coast of Hainan.

OLE is a statistical method used to estimate extinction dates for species based on sighting records. This approach is particularly useful when dealing with incomplete or sparse observational data, which is common in the study of rare or declining species [3,30]. OLE works by analysing the timing of the last few sightings of a species to calculate the most likely time it went extinct [31,32]. The method assumes that sightings follow a random process, often modelled as a Poisson process, where the probability of observing a species decreases over time as its population declines. By fitting a model to the distribution of sighting intervals, OLE generates an estimate of the extinction date along with confidence intervals (CIs). One of the advantages of OLE is its robustness in handling uneven sighting records, allowing it to provide reliable estimates even when data are limited or irregular. It has been widely applied in conservation biology, palaeontology and other fields where understanding the timing of extinction events is critical for decision-making and historical analysis. Although this method relies on the implicit assumption that recording effort never falls to zero [32], the coastal waters of the South China Sea have had an extremely high human population, and fishing effort has been essentially continuous throughout the time period of interest. In addition, to account for estimation uncertainty more comprehensively, we applied a non-parametric bootstrap procedure. Specifically, we resampled the observed sighting years with replacement and recalculated OLE estimates 1000 times for each region to generate a distribution of extinction year estimates. This approach enables the construction of empirical CIs that do not rely on assumptions of symmetry. Given that sighting data are often sparse and clustered, it is unreasonable to maintain a symmetric distribution of estimation error. The bootstrap method provides a more realistic representation of uncertainty under these conditions, enhancing the robustness of extinction date inference. We followed Solow’s [31] implementation of the technique using the ‘sExtinct’ package and performed bootstrap analyses in R 4.1.0 [33].

## 3. Results

We collected 1486 valid questionnaires from all 11 coastal provinces of mainland China in 2024, with 841 samples from possible dugong distribution areas in Fujian, Guangdong, Guangxi and Hainan. The average age of informants in these four provinces was 56.55 ± 13.02 years (range = 22–91, *n* = 833) and fishing experience of 28.70 ± 13.93 years (range = 1–70, *n* = 830). The surveyed fishers were predominantly older adults, with 131 aged over 70 and most between 41 and 70 years (169 aged 41−50, 235 aged 51−60 and 184 aged 61−70), compared to only 17 aged ≤ 30. Most fishers had 11−40 years of fishing experience (189 reporting 11−20 years, 212 with 21−30 years and 167 with 31−40 years), while fewer had 10 years or less (105), 41−50 years (114), or over 50 years (43). These data highlight an older informer group with extensive fishing experience, knowledgeable about marine animal distributions in fishing areas. Analysis of boat length shows that the fishing vessels were generally small, with an average length of 18.61 ± 12.66 m (range = 3–100 m, *n* = 828); most were under 20 m (257 ≤ 10 m, 313 between 11 and 20 m), suggesting that they primarily operate in coastal waters that overlap with dugong habitats.

Only 6.54% of informants (*n* = 55) reported dugong sightings in the 2024 survey. One report was excluded due to its location in Vietnam, and two others were excluded as the informants could not recall the exact sighting year, leaving 52 records with confirmed last sighting dates. Only one fisherman reported a recent dugong sighting, stating that in September 2021, he saw a sea cow weighing over 150 kg that had been caught in a trawl net in Dianbai, Maoming (table 1). The informer described that ‘The animal had a wide mouth and was feeding on seagrass’. Apart from this recent report, all other sightings (98.08%, *n* = 51) dated to or before 2008 when the last confirmed dugong stranding occurred in Wenchang, with the average last sighting date being in 1983 ± 14. In Beihai, Guangxi, only 29.63% (*n* = 8 out of 27 records) of informants reported sightings after the establishment of the national dugong reserve in 1986, suggesting that dugongs were already difficult to find by that time. Analysis of informant age further indicates that direct sighting experience was predominantly reported by older individuals, with a mean age of 61.89 ± 10.78 years (range = 44–83, *n* = 45), which is 5.34 years older than the overall average age.

**Table 1.** Sighting records of dugongs reported by informers in some Chinese waters were never recorded before.

fisher	city	last sighting year	last sighting location	description
1	Fuzhou	1980	Southern Taiwan Strait	The mouth and head look like a cow.
2	Shantou	1975	Nanpeng Islands	He identified the animal in the picture as a sea cow. In the 1970s, he sighted dugongs around the coastal waters of the Nanpeng Islands. Observing from the island, he saw one or two individuals grazing on seagrass. It was a rare sight—he only encountered them two or three times in total, back when the seagrass was still abundant.
3	Shantou	1997	Caoyu Island	I saw a sea cow when I was 19. It weighed several tons and had a flat nose. We called it mermaid. I spotted it eating seagrass around the waters Caoyu, a small island that used to have an abundance of seagrass. There is still some seagrass near the island. That was the only time I saw one.
4	Zhanjiang	2021	Maoming Dianbai	Three years ago, a sea cow weighing over 150 kg was caught by trawling in Dianbai, Maoming, in September. It had black skin and a wide mouth and was feeding on seagrass. The sea cow was released with red strings tied around its neck and flippers, accompanied by the setting off of firecrackers. The catch location featured seagrass beds, but the seagrass has significantly diminished due to declining water quality over the past two years.
5	Zhanjiang	1960	Donghai Island	In June of around 1960, a dugong was captured on the beach at Dongnan Port, and I have not seen one since. In the 1960s and 1970s, there was a large distribution of seagrass around Dongnan and Naozhou Island, but the seagrass significantly decreased after 1978.
6	Zhanjiang	1979	Wailuo Port	Forty years ago, my grandfather caught a sea cow. They called it a seahorse.
7	Zhanjiang	1985	Qishui Harbour	When he saw the photos, he clearly identified it as a sea cow. He mentioned they weigh at least 50 kg and are often found close to shore (they were even seen inside Qishui Port in the 80s). These animals are rare, herbivorous and not commonly seen. When they appear, the water is usually murky because they stir up sediment as they move, making it difficult to see them clearly. The area was once abundant with seagrass, but seagrass beds were destroyed when sand was dredged to build the port. In the 1980s, some sea cows were caught and sold locally as food.
8	Wenchang	1975	Qixingling waters	In the 1970s, I saw a pair of dugongs just a few dozen metres offshore at Qixingling. One was killed with a cannonball, while another managed to escape. It weighed about 350 kg and had hard white fur on its back. During the 1970s, there was a lot of seagrass in the nearby waters, and dugongs came to feed on it. However, due to private aquaculture discharging wastewater into the sea, the once-abundant seagrass has disappeared.
9	Wenchang	1982	50 km offshore	In the early 1980s, I saw a dead sea cow floating on the marine surface about 50 km from the local shore. Due to aquaculture reasons, seagrass has decreased significantly. There are still sea cows in the Indian Ocean and the Pacific Ocean.
10	Dongfang	2005	Weizhou Island	His description matched the basic characteristics of dugongs. The last sighting was in lunar August 2005, when he observed dugongs with both mother and calf, surfacing to breathe while crabbing near Weizhou Island. Weizhou Island was undeveloped at that time and surrounded by extensive seagrass beds and abundant fish.
11	Ledong	1980	Yinggehai	Their heads look like hippos. I saw people catch them before. I also ate their meat, which tastes similar to pork.
12	Sanya	1970	Yalong Bay	I saw a sea elephant twice at Yalong Bay. The last one was killed with a fish cannon in 1970. It weighed 300 kg and was about 3 m long. The meat was similar to pork with both fatty and lean parts.
13	Lingshui	1975	nearby waters	He saw dugongs only once in the summer of 1975. They referred to it as a sea elephant.

We combined sighting data from 2019 and 2024 to explore the historical distribution of dugongs in China. The northernmost record comes from Pingtan Island near Fuzhou, where an 82 year old fisherman recalled seeing a dugong in the southern Taiwan Strait in 1980, although he could not remember the exact location. A total of 21 informants (24%) reported historical sightings in three cities of Guangdong: Shantou (*n* = 4), Maoming (*n* = 1) and Zhanjiang (*n* = 16). Among the sightings in Zhanjiang, nine occurred in the western waters and seven in the east (figure 1A). The largest number of dugong sightings was recorded in Guangxi (*n* = 44, 38%, figure 1B), nearly all located in the waters around Beihai with only one exception in Fangchenggang and another near Weizhou Island in 2005. In Hainan (*n* = 20, 23%), most sightings were reported within the previously known northwestern range (*n* = 16), including Haikou, Wenchang, Chengmai, Danzhou, and Dongfang, but some were also recorded in the southern cities of Ledong, Sanya, and Lingshui, where no historical dugong distribution has been documented (table 1).

To investigate the spatio-temporal extinction dynamics of the dugong population in China, we conducted a comparative analysis of the last sighting years with other historical records (*n* = 134). With a normal data distribution (Shapiro–Wilk test, *p* = 0.212) and homogeneous variances across groups (Bartlett’s test, *p* = 0.785), we applied an ANOVA to test the differences across provinces, and no significant differences were found (F = 1.946, *p* = 0.150). Specifically, the mean ± s.d. sighting years were 1990 ± 20 (range = 1957–2021, *n* = 18), 1983 ± 14 (range = 1960–2013, *n* = 41) and 1990 ± 14 (range = 1970–2010, *n* = 16) in Guangdong, Guangxi and Hainan, respectively (figure 1C). This result is further supported by interval estimation, with 95% CIs for the last sighting years in six regions partially overlapping. Based on all available sighting records combined, the overall extinction year was estimated to be 2023 (95% CI: 2021−2026). Furthermore, OLE with asymmetric interval estimation using the bootstrap method suggested the extinction year was 2035 in eastern Guangdong (zone 1, 95% CI: 2005−2047), 2025 in eastern Zhanjiang (zone 2, 95% CI: 2006−2037), 2018 in western Zhanjiang (zone 3, 95% CI: 1994−2031), 2014 in Guangxi (zone 4, 95% CI: 2007−2017), 2004 in western Hainan (zone 5, 95% CI: 1993−2008) and 2012 in eastern Hainan (zone 6, 95% CI: 1990−2018). The overlapping 95% CIs for the estimated extinction years across different regions indicate that there is no significant difference in the extinction timing among these areas (figure 1D). Thus, there is no evidence of population fragmentation or range contraction over a large spatial scale during the population decline.

We also gathered data on factors contributing to dugong population decline based on the 2024 survey, such as seagrass bed destruction, capture incidents and consumption. The findings revealed that 24 reports (43.64%) involved dugong deaths, 20 (36.36%) described live individuals, while the remaining 11 did not specify the animal condition. Additionally, 29 informants (52.73%) reported seagrass beds in or near dugong sighting locations; however, nearly all these informants (*n* = 25, 86.21%) noted that the previously abundant seagrass had either disappeared or significantly declined. Moreover, 25 informants (45.45% of sighting informers, 2.97% of interviewed respondents) reported having previously caught dugongs, 15 (27.27% of sighting informers, 1.78% of all respondents) had eaten their meat and 2 had witnessed dugongs being killed with explosives.

## 4. Discussion

While LEK is widely promoted by international conservation organizations such as the IUCN, its application in population monitoring is sometimes met with concerns regarding data consistency, recall bias and integration with scientific methods that can lead to inaccuracies in species sightings, population estimates and trends over time [24,34]. For example, informers’ descriptions of dugong characteristics may be inaccurate in aspects such as skin colour, body size, local names and head shape, requiring careful evaluation using multiple lines of evidence. LEK data may also lack the spatial and temporal precision typically associated with systematic scientific surveys, making it challenging to establish accurate population baselines or detect subtle trends [35]. Furthermore, the anecdotal nature of LEK can introduce data variability due to local beliefs or practices [36]. However, the use of LEK in endangered species research has been noted as essential and is sometimes the only tool for assessing the status and trends of highly scarce populations [3]. Our study reaffirms that for species like the dugong, which once had a wide distribution but now exists in critically low numbers in China, conventional methods such as high-wing aircraft, boat- or helicopter-based visual surveys and passive acoustic monitoring face significant limitations. In such cases, LEK surveys that draw on the decades of fishing knowledge held by local fishers offer important complementary value. While not a replacement for field-based surveys, LEK can be especially advantageous for detecting rare or past occurrences that may go unnoticed by conventional methods. Moreover, LEK is uniquely suited for understanding long-term changes in population distribution over decadal time scales. For the Chinese dugong, which has become functionally extinct without having been the subject of comprehensive scientific surveys [16,37], LEK provides invaluable information for reconstructing historical population conditions and offers critical insights into past population dynamics.

The term functional extinction refers to the permanent failure of reproduction or recruitment within a population [38]. For marine mammals, the minimum viable population is generally estimated to be between 10 and 30 individuals [39]. The present findings confirmed the conclusion of the functional extinction of dugongs in China. This conclusion was originally drawn from the 2019 survey of fishers across all potential dugong distribution areas in China, supported by historical sighting records and seagrass bed distribution data [16]. The conclusion has been widely accepted by both the academic community and the public [25,37], with the IUCN Red List assessment group suggesting updating the dugong status in China to critically endangered (CR) with the same level as the Japanese subpopulation [40]. However, some scepticism remains, with suggestions that dugongs may still inhabit the nearly 2000 km coastline of the South China Sea, warranting further research. A typical case occurred on 25 March 2025, when a dugong was incidentally captured and subsequently released by fishers in Yilan County (24.50° N, 121.84° E), Taiwan, China. Our results showed that all reported sightings of dugongs originated from the provinces of Guangxi, Guangdong and Hainan, with no historical records from the eight provinces north of Guangdong. Apart from a suspected trawling capture of a dugong in Dianbai, Maoming, in 2021 (a site with historical stranding records), there have been no other recorded reports by informers since the last stranding in 2008 with an average last sighting date over three decades ago (1983 ± 14). Although the 2019 survey highlighted recent dugong sightings near Shantou [16], prompting focused efforts in the 2024 questionnaire survey in nearby waters. Despite this, responses from 49 experienced fishers in Shantou and 47 in the neighbouring city of Zhangzhou confirmed the absence of a resident dugong population in the region. While two fishers reported dugong sightings in 1975 (three times) and 1997 (once), the rarity and low frequency of these reports suggest these were likely transient or residual individuals. Regrettably, these findings all indicate that there is no evidence to suggest that more than 30 dugongs remain in Chinese waters, despite the possible presence of a few wandering individuals, which meets the criteria for functional extinction.

Our results significantly expand the known historical range of dugong in Chinese waters. East Asia was once an important habitat for dugongs [41,42]. In Japan, their northernmost distribution reached southern Kyushu, with the Ryukyu Islands being the most significant habitat [37,40]. In China, Wang & Sun suggested that the primary areas of dugong distribution are in the coastal waters of Beibu Gulf and western Guangdong, with the northernmost limit reaching southern Taiwan [21]. The recent bycatch incident in Yilan County indicates that dugongs can also be recorded in northeastern Taiwan, although a resident population may have never existed on this island historically [37]. In addition, Wang & Sun did not provide credible records of dugong in Zhanjiang and explicitly noted the absence of sightings in Xuwen. Our investigation uncovers multiple historical records of dugong sightings in both the western and eastern waters of Zhanjiang (figure 1A), offering concrete evidence for the historical distribution of dugongs in this area. Meanwhile, they also speculated that dugongs in Maoming and Yangjiang may have strayed from the Beibu Gulf through the Qiongzhou Strait. Given the frequent sighting reports of dugongs in Zhanjiang, the occasional sightings in these two cities also likely indicate the historical presence of a small resident population. Our study also shows that the eastern Hainan Island was historically part of the dugong range, confirming the accuracy of the previous distribution map [41,42]. A notable sighting incident occurred in 2008, when a fresh dugong carcass was found stranded in Wenchang, sparking discussion about whether a stable dugong population existed in the eastern waters of Hainan Island [43]. Due to this being the only confirmed record in the area, it was assumed that this individual had wandered from another location, such as the northern Philippines. Our results provide new evidence supporting the possible existence of a small population or an area where dugongs were frequently sighted in eastern Hainan historically.

Although large populations of dugongs are found in coastal continental habitats of Australia [44,45], coastal islands in East Africa, the Arabian Gulf and Southeast Asia with seagrass beds are also considered the preferred habitats for dugongs [46–48]. For example, in Southeast Asia, the Mersing Archipelago in Malaysia, the Palawan Islands in the Philippines and the Maluku Islands in Indonesia were identified as important habitats for dugong [47,48]. Although many Chinese provinces along the South China Sea are home to numerous islands of varying sizes and distances from the mainland, there have been no confirmed reports of dugong populations in these islands to date. Two informants provided records of dugongs around Weizhou Island in the Beibu Gulf (table 1). The first one was a 62 year old fisherman from Beihai, who recounted hearing that dugongs were once abundant around the island. The second one was a 56 year old fisherman from Dongfang, who reported personally observing dugongs around Weizhou Island on several occasions, with his last sighting in 2005 when a group of dugongs surfacing to breathe were observed in a seagrass bed. This finding offers evidence of historical continuity in dugong populations from China to Vietnam and nearby islands. Additionally, historical sightings on Nan'ao Island (23.40° N) and the Nanpeng Islands (23.27° N) near Shantou suggest that dugongs once inhabited these regions as well. These records extend the northernmost known range of dugongs in mainland China, which was previously thought to be Yangjiang (21.49° N) in the western Guangdong Province. Simultaneously, the continuity of dugong distribution between Taiwan and eastern Guangdong challenges the hypothesis proposed by Wang & Sun [21]. They argued that dugongs in Taiwan were isolated from those in mainland China, and thus might have migrated northward from the Philippines. The sighting records from Shantou suggest that dugong populations in Taiwan and mainland China were likely connected rather than completely isolated.

It is generally believed that the species extinction process involves a gradual geographical range contraction accompanied by population abundance decline, eventually leading to the species disappearing generally near the centre of the historical range [49,50]. Although this pattern may vary depending on species-specific traits and external drivers [49], this dynamic property of range collapse provides critical time for population monitoring and conservation efforts, offering the last opportunity to save a species from the extinction vortex. As a result, the geographical range and its rate of change have become important criteria in assessing population status, e.g. the IUCN Red List. However, this extinction process was challenged by the study on some marine mammals. Research on the baiji revealed that the population decline was not associated with any significant range contraction across the middle-lower Yangtze drainage, even in the decade leading up to the species’ probable global extinction [3]. Thus, the extinction risk of the baiji appeared unrelated to range contraction or fragmentation. Our study on the last sighting years in different regions also indicates that dugongs disappeared almost simultaneously across their historical range in China, without undergoing obvious range contraction. This may be attributed to the ability of dugongs to undertake large-scale movements in search of mates when population sizes are low or in search of food when seagrass beds are degraded. As a result, sightings may still occur across scattered locations, even in the final stages of population decline. This finding once again challenges the hypothesis that range contraction are prior indicators of species extinction. It is thus recommended to further study the population decline and range contraction of marine megafauna to determine whether this is a widespread phenomenon or only occurs in limited species such as the dugong and baiji with strong migratory abilities.

Although dugongs have been recorded in Chinese waters for at least several centuries [18], their abundance has never been estimated. We infer there were likely several hundred dugongs inhabiting Chinese waters at the time of the founding of the People’s Republic of China in 1949. This inference is based on the fact that 257 dugongs were hunted for food between 1958 and 1976 according to unpublished fisheries records [16]. These figures are based on centralized hunting statistics from fisheries management, which likely exclude many cases of sporadic dugong hunting by individual fishermen. While waters around Beihai are generally considered the primary habitat of dugongs, it had 540 hectares of seagrass beds by 2005 [51] and only 0.51 hectares in 2014 [52]. This limited distribution of seagrass alone could not have sustained a population of several hundred dugongs. Our survey revealed that over half of the informants reported seagrass beds in or near locations where dugongs were sighted, suggesting that seagrass beds were once widely distributed across the coastal waters of the South China Sea. Therefore, the historical dugong population was likely supported by extensive seagrass beds along the Beibu Gulf and other coastal areas of the South China Sea. Unfortunately, nearly all informants (86%) noted that the once-abundant seagrass had either disappeared or significantly declined, a finding consistent with the latest surveys of China’s seagrass beds, which indicate that 80% of historical distribution has vanished since the 1970s [28]. This suggests that the large-scale destruction of seagrass beds has been a major factor contributing to the functional extinction of dugongs in China, in addition to the well-documented impact of human hunting [17].

Currently, dugong populations have gone extinct in multiple countries/regions, including Comoros (outside Mohéli), Mauritius (including Rodriguez) and the Seychelles (outside Aldabra) [53]. Although recent studies estimate the total dugong population to be around 1 65 000 individuals in Australia [44], only six other regions (Bazaruto Bay in Mozambique, the Red Sea coast of Saudi Arabia, the southeastern Arabian Gulf, Trang in Thailand, the Mersing Archipelago in Peninsular Malaysia and New Caledonia) are known to support dugong populations exceeding 100 individuals [53]. The outlook for dugong conservation worldwide remains highly precarious. The decline of the dugong population in China with failed conservation lessons can provide valuable warnings for other nations. In 1986, a national-level dugong sanctuary was established in Beihai, and dugongs were also included in the national list of first-class protected animals in 1988, with hunting being prohibited. However, this intervention came too late; according to surveys, the population had already collapsed by then, making dugong sightings exceptionally rare, even for experienced local fishers. The Chinese government also considered establishing a sanctuary in western waters of Hainan and conducted preliminary field studies in the early 2000s [23], but it was also too late as dugongs likely disappeared at that time. Anyway, the extinction of the Chinese dugong population illustrates the extreme vulnerability of this species. Within just 20−30 years, at least hundreds of individuals were wiped out due to human activities, disappearing almost simultaneously across entire range. This starkly demonstrates the need for other countries to act swiftly in implementing conservation measures and to rigorously protect seagrass habitats while curbing hunting and accidental capture in dugong habitats.

## Data Availability

The data are published in the article and provided as electronic supplementary material [54].
